# Crack-resistant Al_2_O_3_–SiO_2_ glasses

**DOI:** 10.1038/srep23620

**Published:** 2016-04-07

**Authors:** Gustavo A. Rosales-Sosa, Atsunobu Masuno, Yuji Higo, Hiroyuki Inoue

**Affiliations:** 1 Institute of Industrial Science, The University of Tokyo, 4-6-1 Komaba, Meguro, Tokyo 153-8505, Japan; 2 Japan Synchrotron Radiation Research Institute, 1-1-1 Kouto, Sayo, Hyogo 679-5198, Japan

## Abstract

Obtaining “hard” and “crack-resistant” glasses have always been of great important in glass science and glass technology. However, in most commercial glasses both properties are not compatible. In this work, colorless and transparent *x*Al_2_O_3_–(100–*x*)SiO_2_ glasses (30 ≤ ***x*** ≤ 60) were fabricated by the aerodynamic levitation technique. The elastic moduli and Vickers hardness monotonically increased with an increase in the atomic packing density as the Al_2_O_3_ content increased. Although a higher atomic packing density generally enhances crack formation in conventional oxide glasses, the indentation cracking resistance increased by approximately seven times with an increase in atomic packing density in binary Al_2_O_3_–SiO_2_ glasses. In particular, the composition of 60Al_2_O_3_•40SiO_2_ glass, which is identical to that of mullite, has extraordinary high cracking resistance with high elastic moduli and Vickers hardness. The results indicate that there exist aluminosilicate compositions that can produce hard and damage-tolerant glasses.

Strong glasses combining the mechanical attributes of being “hard” and “crack-resistant” are required in many technological fields including construction, transportation, and electronics. However, the inherent brittleness of glasses limits their applications. The simultaneous realization of high hardness and crack resistance has always been an important issue in glass science and technology.

The hardness of glasses is often evaluated by Vickers hardness *H*_V_, which is estimated from the size of indentation imprints. Vickers hardness in conventional oxide glasses can be predicted with relative good accuracy using compositional and structural parameters. Yamane *et al*. proposed an equation for silicate glasses in which *H*_V_ is proportional to the square root of the product between the bulk modulus *K*, shear modulus *G*, and the average single-bond energy between the component cations and oxygen^1^. Positive linear trends have been found between Vickers hardness and Young’s modulus *E*^2^,^3^. According to the Makishima and Mackenzie model, the Young’s modulus *E*, bulk modulus *K*, and shear modulus *G* of glasses are related to both the dissociation energy per unit volume of the components and the atomic packing density of the glass^4^,^5^. In case of alkali–alkaline earth silicate glasses, it was found that glasses with more packed structures had higher values of hardness and the elastic modulus^6^. Al_2_O_3_–*R*_2_O_3_ and Al_2_O_3_–SiO_2_–*R*_2_O_3_ glasses (*R*: Y, Sc, or Ta) are among the hardest oxide glasses (*H*_V_ up to 9.5 GPa) with high elastic moduli (*E* up to 169 GPa)^7^,^8^,^9^,^10^,^11^,^12^,^13^. This is attributed to their high atomic packing density and high dissociation energy unit volume of components. Based on successful theoretical temperature-dependent constraints, a recent model was proposed in which *H*_V_ is proportional to the number of bond-stretching and bond-bending constraints at room temperature in oxide glasses^14^,^15^. These results suggest that a hard glass with high elastic moduli require a high atomic packing density and high bonding energy of components.

”Crack-resistant” or low –brittleness glasses should be resistant to surface damage or fracture. An effective way to increase the cracking resistance of glasses is chemical or physical strengthening of the glass surface by creating a compressive stress layer at the surface that prevents crack initiation and propagation. The thickness of the compressive stress layer is limited to a few microns beneath the surface without affecting the interior physical properties of the glass^16^,^17^. Low-brittleness glasses have been designed by adjusting the chemical composition to enhance the glass deformation ability. Sehgal and Ito developed a less-brittle (LB) soda–lime–silica glass with remarkably high indentation cracking resistance by tuning the molar volume of the glass^18^. Also, Sehgal and Ito showed that the LB glass displayed a lower brittleness and higher indentation fracture toughness than normal soda-lime silicate glass. Gross *et al*. synthesized a 10CaO•10Al_2_O_3_•80SiO_2_ glass with high-crack initiation load and fictive temperature-independent elastic modulus^19^. Morozumi *et al*. fabricated a sodium aluminoborosilicate glass with a high intrinsic cracking-initiation load^20^. These crack-resistant glasses can release the stress without cracking by atom displacements, resulting in plastic deformation mainly by densification and, to a certain extent, by shear deformation^21^,^22^,^23^,^24^,^25^. Because of their high molar volume and low atomic packing density, in these glasses atoms can easily be compacted by force. Moreover, crack-resistant glasses are characterized for having low elastic moduli and Vickers hardness (*H*_V_: approximately 5–6 GPa and *E*: approximately 60–70 GPa). Therefore, it is accepted that “hard” and “crack-resistant” are not achieved simultaneously in conventional oxide glasses because high packing density is a characteristic of hard glasses. On the contrary, in this paper, we show that alumina-rich Al_2_O_3_–SiO_2_ binary glasses exhibit both high hardness and cracking resistance. Furthermore, we investigated the correlation among atomic packing density, elastic moduli, and indentation of the binary aluminosilicae glasses.

## Results

The Al_2_O_3_–SiO_2_ binary glass system is important in geosciences, glass-ceramics, and is a base composition for fabricating multiple commercial glasses. It has been widely studied owing to its interesting structure and phase-separation phenomena. In particular, binary aluminosilicate glasses with more than 30 mol% of Al_2_O_3_ are difficult to fabricate in the bulk form by conventional melting processes because of their low glass-forming ability and high melting temperatures. Al_2_O_3_-rich glasses have been prepared by splat quenching, flame spraying, roller quenching, sol-gel, and containerless processing^26^,^27^,^28^,^29^,^30^,^31^,^32^,^33^. Most techniques produce thin flakes or small particles, whereas containerless processing has the advantage of producing bulk glasses by solidification without heterogeneous nucleation at the surface of the melt. Recently, it has been reported that TiO_2_-, Nb_2_O_5_-, WO_3_-, and Al_2_O_3_-based glasses without any network formers have been successfully fabricated with this method^34^,^35^,^36^,^37^,^38^,^39^. Weber *et al*. obtained Al_2_O_3_–SiO_2_ glasses with Al_2_O_3_ content up to 67 mol% by aerodynamic levitation (ADL); however, the diameter of the glass samples was at most 1 mm^33^. In this work, we used the ADL technique for fabricating bulk Al_2_O_3_–SiO_2_ glasses with high alumina content.

**Figure 1:**
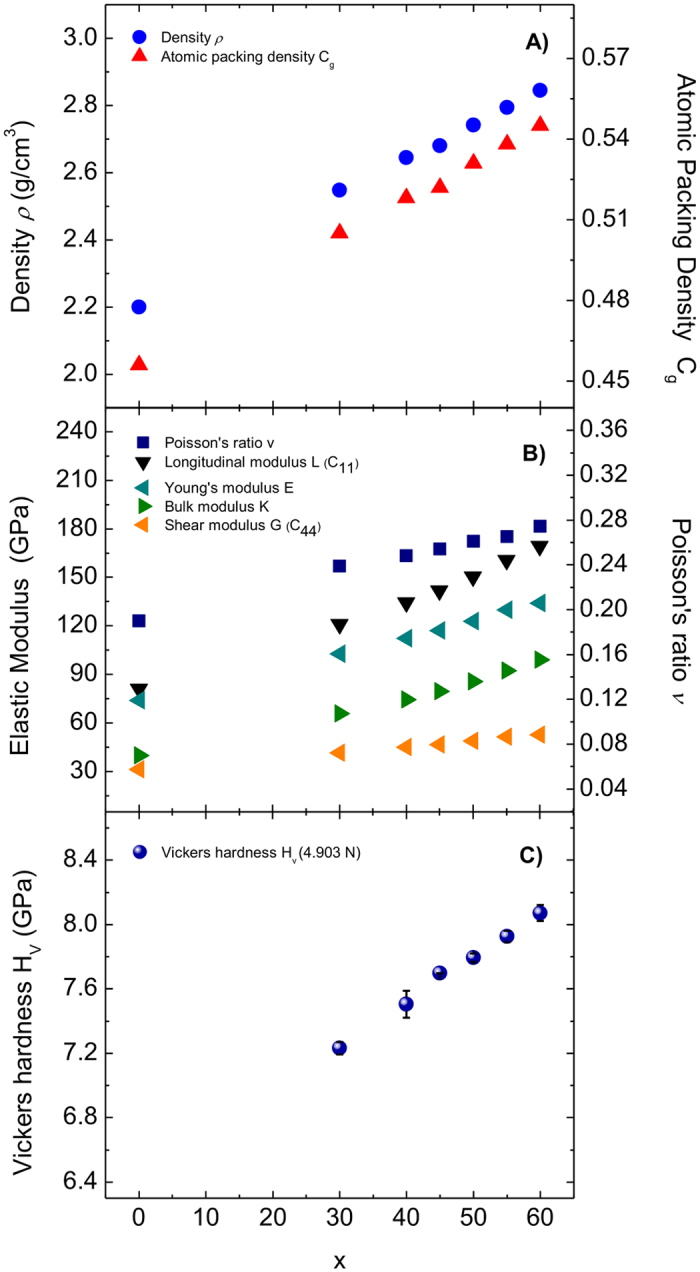
Composition dependence of (A) the density *ρ* and the atomic packing density *C*_g_, (B) elastic moduli: *L* (*C*_11_), *K*, *G* (*C*_44_), *E* and *ν*, and (C) Vickers hardness *H*_V_ for the *x*Al_2_O_3_–(100–*x*)SiO_2_ glasses. The data for SiO_2_ glass are shown at *x* = 0.

Transparent and colorless *x*Al_2_O_3_–(100–*x*)SiO_2_ glasses were obtained in the range of 30 ≤ *x* ≤ 60. At *x* ≥ 60, mullite and α-Al_2_O_3_ directly crystallized from a melt. The properties of the glasses are summarized in Table 1. Figure 1A shows the composition dependence of density *ρ* and atomic packing density *C*_g_. Both *ρ* and *C*_g_ increase linearly with Al_2_O_3_ content and are in good agreement with the previous results obtained from roller-quenched and splat-quenched amorphous flakes^28^,^29^. The glasses become more packed and rigid as alumina content increases. Figure 1B shows that all the elastic moduli increase linearly with *x*. Young’s modulus *E* increases from 102.9 GPa to 134.2 GPa, bulk modulus *K* increases from 65.6 to 99.0 GPa, shear modulus *G* increases from 41.5 GPa to 52.7 GPa, and Poisson’s ratio ν increases from 0.239 to 0.274 with increasing alumina content. The values of *ρ*, *C*_g_ and elastic moduli seem to extrapolate linearly to the pure SiO_2_ composition. In Fig. 1C, the Vickers hardness *H*_V_ increases monotonically with increasing Al_2_O_3_ content from 7.23 to 8.07 GPa in agreement with the elastic moduli trends. The increase in elastic moduli and hardness of *x*Al_2_O_3_–(100–*x*)SiO_2_ glasses with increasing *x* follows the expected relation between elastic moduli and hardness as well as the atomic packing density and dissociation energy per unit volume of the components. The increase in Al_2_O_3_ content enhances the atomic packing density and total dissociation energy of the glass because of the large dissociation energy of Al_2_O_3_ per unit volume (*G*_Al2O3_ = 131 kJ/cm^3^) compared with SiO_2_ (*G*_SiO2_ = 68 kJ/cm^3^)^40^. As a result, the glass with the highest Al_2_O_3_ content (*x* = 60) with mullite composition shows the highest elastic moduli and Vickers hardness. These values are comparable to hard glasses, such as *R*_2_O_3_–SiO_2_–Al_2_O_3_ or CaO–SiO_2_–Al_2_O_3_ glasses, and are much larger than those of crack-resistant silicate or borosilicate glasses^7^,^8^,^9^,^10^,^11^,^12^,^13^,^18^,^19^,^20^,^21^,^22^,^23^,^24^,^25^.

**Figure 2:**
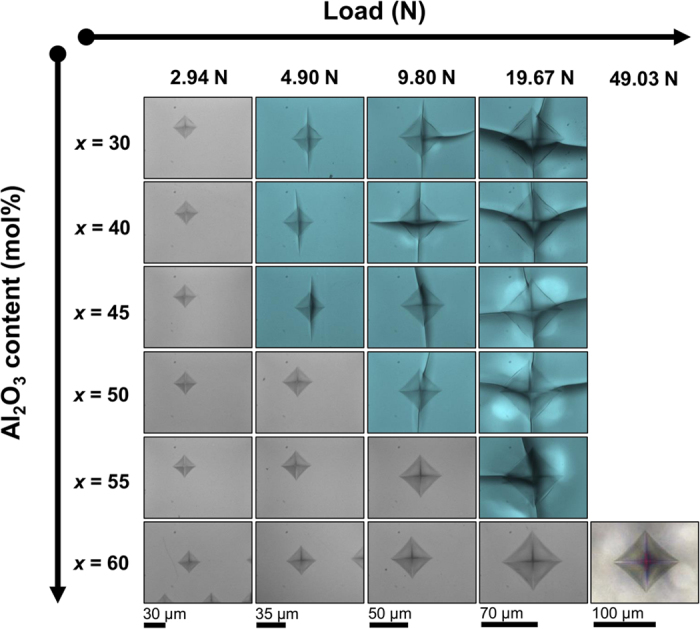
Vickers indentation imprints for the *x*Al_2_O_3_–(100–*x*)SiO_2_ glasses. Blue-shaded photographs show the cracked samples. Gray-shaded photographs show the non-cracked samples. Each indentation imprint represents more than 50% of the indentations.

**Figure 3:**
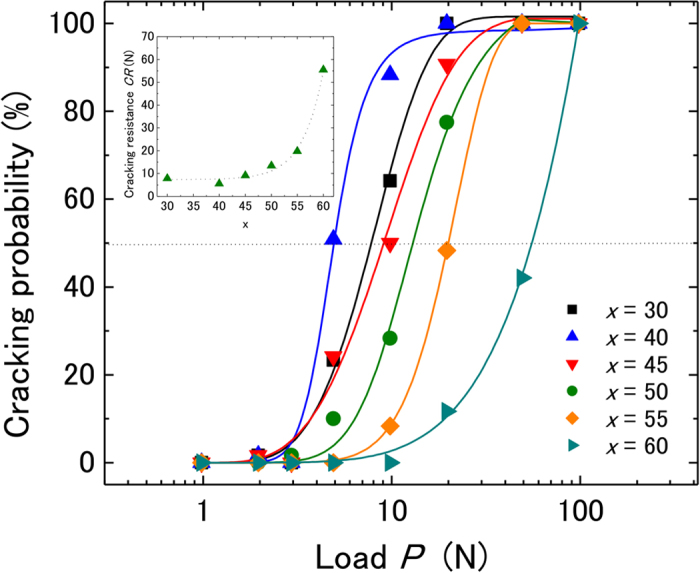
Cracking probability curves for the *x*Al_2_O_3_–(100–*x*)SiO_2_ glasses. The inset shows the composition dependence of the cracking resistance.

In order to rule out any surface effect in the glasses obtained by the levitation system we compared the hardness and indentation imprints of pure SiO_2_ glass as prepared by the aerodynamic levitation system with a reference SiO_2_ glass (Edmund optics). The values of indentation hardness *H*_IT_ were 8.69 ± 0.08 GPa and 8.69 ± 0.07 GPa for the levitation SiO_2_ glass and the reference SiO_2_ glass respectively. Also, the indentation imprints displayed the same cracking patterns with cone cracks typical of anomalous glasses and similar cracking frequency. These results suggest that there is no apparent surface effect on the indentations properties of the glasses obtained through the levitation system. Figure 2 shows the Vickers imprints on *x*Al_2_O_3_–(100–*x*)SiO_2_ glasses for various loads. It is noted that with increasing Al_2_O_3_ content, the glasses become more resistant to radial cracking (observed at the imprint corners) although the atomic packing density increased. Normal (radial cracks) behavior is observed in all samples for *x* = 30, 40, 45, 50, and 55, whereas no cracks were observed in more than 50% of the indentation imprints for glasses with *x* = 60 even using an indentation load of 49.03 N. To quantify the resistance to fracturing, cracking probability curves are shown in Fig. 3. The data were fitted by using a sigmoid function. From the fitting curve, the loading force required to generate a 50% cracking probability or 2 radial cracks in average (cracking resistance *CR*) was estimated. As shown in the inset, *CR* increases drastically at *x* ≥ 50. The *CR* value of 55.4 N for the *x* = 60 glass is considerably larger than those of the crack-resistant glasses. For example, Asahi’s LB glass had an indentation cracking resistance of approximately 30 N measured in nitrogen (N_2_), a 80SiO_2_•10Al_2_O_3_•10CaO glass had *CR* of approximately 10 N under N_2_, an aluminoborosilicate commercial glass had *CR* of 11 N at 30% relative humidity, and a 80SiO_2_•15Na_2_O•5CaO glass had 10 N *CR* at 30% relative humidity^18^,^19^,^22^,^41^. It is well known that the indentation cracking resistance in silicate glasses strongly depends on the atmosphere during measurements and that humidity decreases *CR*^42^,^43^. Therefore, it is likely that the *CR* of the *x* = 60 glass will increase when measured in a nitrogen atmosphere. The large *CR* of the *x* = 60 glass is comparable to chemically strengthened soda lime glass^17^.

## Discussion

Oxide glasses with large *CR* usually have enough free space to dissipate the mechanical stress owing to densification (compaction) and to lesser extent shear deformation. Therefore, large *CR* in oxide glasses should correlate to open structure (less-packed)^18^,^21^,^22^,^25^. Rouxel *et al*. suggested the use of Poisson’s ratio ν as to describe the available free volume in glasses^44^. Based in hydrostatic compression experiments, the simple equation Δ*ρ*/*ρ*_o_ = 150∙exp^(−13∙*ν*)^ was proposed, where Δ*ρ*/*ρ*_o_ is the maximum relative density change, *ρ*_o_ is the initial density, and Δ*ρ* is the density change owing to hydrostatic stress^44^. In case of *x*Al_2_O_3_–(100–*x*)SiO_2_ glasses, the Δ*ρ*/*ρ*_o_ decreases from 6.71% to 4.25% with increasing *x* from 30 to 60. This suggests that increasing the Al_2_O_3_ content may reduce the ability for densification therefore decreasing the cracking resistance. Moreover, Sellapan *et al*. showed that the radial/median cracking ability of silicate and borosilicate glasses may be classified in relation to their Poisson’s ratio ν as: resistant 0.15 ≤ ν ≤ 0.20, semi-resistant 0.20 ≤ ν ≤ 0.25, and easily damaged 0.25 ≤ ν ≤ 0.30^25^. Based on Poisson’s ratio, the Al_2_O_3_–SiO_2_ glasses should change from semi-resistant to easily damaged as the Al_2_O_3_ content increases. However, it is observed that *CR* increases with alumina content. The increase in the *CR* of binary aluminosilicate glasses is the opposite of what is expected. Apparently, other mechanism to prevent crack initiation should be taken in consideration.

Shear deformation is an alternative mechanism in conventional oxide glasses. In oxide glasses, such deformation likely occurs in the vicinity of atoms that are weakly bonded owing to the existence of non-bridging oxygen, as observed in sodium silicate glasses^45^. In case of binary Al_2_O_3_–SiO_2_ glasses, non-bridging oxygens were not confirmed by XPS measurements^46^. Thus, shear deformation owing to the movement of non-bridging oxygens is rather unlikely. Shear deformation will also occur in borate glasses where easy-slip units such as boroxol rings exist or where BO_3_–BO_4_ species exchange occurs under applied stress^47^. Recently, similar shear deformation processes have been proposed to occur in densified silica glass^48^,^49^. As in the case of BO_3_ and BO_4_ units in borate glasses or SiO_4_, SiO_5_ and SiO_6_ units found by molecular dynamics in densified silica glass, different structural units, such as AlO_4_, AlO_5_, and AlO_6_ have been observed in Al_2_O_3_–SiO_2_ glasses. ^27^Al Magic Angle Spin (MAS) NMR and molecular dynamics (MD) simulations have shown that alumina-rich Al_2_O_3_–SiO_2_ glasses contain high quantities of distorted AlO_5_ species (about 30–49% of Al sites) apart from AlO_4_ and AlO_6_^32^,^33^,^50^,^51^. Moreover, based on oxygen diffusion in melts, it was suggested that AlO_5_ units in Al_2_O_3_–SiO_2_ glasses are trapped in the glass structure in a meta-stable state between the AlO_4_ in liquids and AlO_6_ in crystalline materials like mullite^33^,^51^. In this sense, it is probable that the multiple coordination environments of Al atoms as well as the mid-range structure around these units play a role on the enhancement of the cracking resistance through shear deformation processes. Although the overall mechanism is still not clear it is important to note that shear deformation processes are favored as the packing density and Poisson’s ratio increases which is observed in the studied system as the alumina content increases. Accordingly, there is a possibility that plastic deformation may be aided not only by densification but also by shear deformation processes in Al_2_O_3_–SiO_2_ glasses.

## Conclusion

The elastic properties and indentation of *x*Al_2_O_3_–(100–*x*)SiO_2_ glasses prepared by aerodynamic levitation were investigated. All glasses in the range 30 ≤ *x* ≤ 60 were colorless and transparent. The elastic moduli and hardness increased monotonically with alumina content from *x* = 30 to *x* = 60. The steady increase of the elastic moduli and hardness can be explained by the increasing atomic packing density and the high dissociation energy per unit volume of Al_2_O_3_ compared to SiO_2_. Furthermore, it was found from the indentation imprints that the glass cracking resistance increases with increasing alumina. As a result, alumina-rich Al_2_O_3_–SiO_2_ glasses are strong materials because of their high hardness and high indentation cracking resistance. In particular, the 60Al_2_O_3_•40SiO_2_ glass displayed the highest indentation cracking resistance, elastic moduli, and hardness in the binary system. The increase in cracking resistance cannot be explained only by densification as it is widely accepted for conventional oxide glasses. Thus, it is proposed that the local structure of aluminum atoms as well as the structure around these units may play a role in the increased cracking resistance of alumina-rich Al_2_O_3_–SiO_2_ glasses through shear deformation processes.

## Methods

### Glass synthesis

The glasses were fabricated using an aerodynamic levitation furnace described elsewhere^52^. Alumina (α-Al_2_O_3_) and SiO_2_ powders (99.99% purity) were mixed in stoichiometric proportions of *x*Al_2_O_3_–(100–*x*)SiO_2_ with *x* = 30, 40, 45, 50, 55, 60. The mixed powders were pelletized in a hydrostatic press and then heated in air at 1050 °C for 12 h. Pieces of the crushed pellets were melted at temperatures between 1800 °C and 2000 °C using two CO_2_ lasers in an ADL furnace. Oxygen was used for levitating the melt and for avoiding SiO_2_ evaporation. The levitated melts were solidified by turning off the lasers at a cooling rate of a few hundreds of degrees per second. The diameter of the solidified samples was approximately 2 mm. Glass formation was confirmed by Cu *Kα* XRD (Rigaku, RINT 2000). Fused silica glass (T-4040, Covalent Materials Corp.) was used for reference.

### Density and atomic packing density

The density *ρ* of the glasses was determined using gas pycnometry (Micromertrics, AccuPycII 1340). The atomic packing density *C*_g_ was calculated from the experimental density using the formula *C*_g_ = *ρ*∙Σ(*x*_*i*_*∙V*_*i*_)*/M*, where *M* is the molecular mass of the glass, *x*_*i*_ is the molar fraction of oxide *i*, and *V*_*i*_ is the ionic volume of oxide *i*. The ionic volume is given by *V*_*i*_ = *N*_A_(4/3)π(*mr*_*A*_^3^ + *nr*_*O*_^3^), where *N*_A_ is Avogadro’s number, *m* and *n* are the number of atoms in the *A*_*m*_*O*_*n*_ oxide, *r*_*A*_ is the ionic radius of the cation, and *r*_*O*_ is the ionic radius of oxygen. The coordination number of Al^3+^, Si^4+^, and O^2−^ was assumed 4, 4, and 2, respectively. Shannon and Prewitt ionic radii were used^53^.

### Elastic moduli measurement

The pulse-echo overlap technique was used to obtain the sound velocities of the glass^54^. A 50 μm thick ultrasonic transducer (LiNbO_3_ 10° Y-cut) and a 500-μm thick glass were pasted at opposite corners of a corner-truncated tungsten carbide (WC) block using a conductive epoxy resin. The ultrasonic echoes of the longitudinal (L) and shear (T) waves from the transducer were reflected by the glass and observed using a digital oscilloscope. The longitudinal velocity *V*_L_ and transversal velocity *V*_T_ were determined by dividing the thickness of the samples by the observed travel time of the waves. The longitudinal modulus *L* (*C*_11_) and shear modulus *G* (*C*_44_) were estimated using equations *L* = *ρV*_L_^2^ and *G* = *ρV*_T_^2^. The Young’s modulus *E*, bulk modulus *K*, and Poisson’s ratio *ν* were calculated using equations *E* = *G*(3*L* − 4*G*)/(*L* − *G*), *K* = *L* − (4/3)*G*, and *ν* = (*L* − 2*G*)/(2*L* − 2*G*), respectively. The elastic properties of SiO_2_ glass were also measured for comparison.

### Indentation behavior

In order to rule out any surface effect in the glasses obtained by the levitation system we compared the hardness and indentation imprints of pure SiO_2_ glass as prepared by the aerodynamic levitation system with a reference SiO_2_ glass (Edmund optics). Spherical SiO_2_ samples obtained by aerodynamic levitation were mirror-polished into a disk shape with a thickness of 500-μm. The indentation experiments were made using a dynamic indenter (Shimadzu DUH-211) loaded with a diamond Berkovich 115° indenter in an atmosphere with 60% relative humidity. The loading and unloading rate was set to 70.067 mN/s using a dwell time of 15 s. The indentation hardness was calculated from the equation *H*_IT_ = *F*_max_/*A*_p_, where *F*_max_ is the applied load and *A*_p_ = *h*_max_ − 0.75(*h*_max_ − *h*_r_) is the projected contact area, where *h*_max_ is the maximum penetration at *F*_max_, and *h_r_* is the point of intersection between the tangent of the linear section of the unloading curve and the indentation depth axis. Ten indentations were performed for each SiO_2_ glass sample. The resulting imprints were observed by optical microscopy. For the Al_2_O_3_–SiO_2_ glasses indentation experiments were performed using a Vickers hardness tester at 23 °C and 60% relative humidity. Mirror-polished glasses with approximately 500-μm thickness were used. A Shimadzu DUH HMV-1 Vickers tester and an Akashi AVK-C2 Vickers tester were used for indentation loads below 19.6 N and over 19.6 N, respectively. The dwell time was 15 s. Vickers hardness *H*_V_ was calculated from the diagonal length of the imprints at a load of 4.903 N. To evaluate the cracking resistance *CR* to radial cracks, the number of corners (four) divided the number of cracks for each indentation and the results were averaged by the number of indentation tests. The averaged value is the cracking probability at specific load. Cracking probability curves were obtained by plotting the cracking probability as a function of the loading force. The indentation cracking resistance (*CR*) is defined as the load required for generating two radial/median cracks on average or associated with 50% cracking probability^55^. Three specimens were used for each composition. At least 20 indentation imprints were used to calculate the *H*_V_ and *CR* at each load.

## Additional Information

**How to cite this article**: Rosales-Sosa, G. A. *et al*. Crack-resistant Al_2_O_3_–SiO_2_ glasses. *Sci. Rep*. **6**, 23620; doi: 10.1038/srep23620 (2016).

## Figures and Tables

**Table 1:** Density *ρ*, atomic packing density *C*_g,_ sound velocities, elastic moduli, and indentation properties of *x*Al_2_O_3_–(100–*x*)SiO_2_ glasses including pure SiO_2_ glass.

x	*ρ*(g/cm^3^)	*C*_g_	*V*_L_(km/s)	*V*_T_(km/s)	*L*(GPa)	*E*(GPa)	*K*(GPa)	*G*(GPa)	ν	*H*_V_(GPa)	*CR*(N)
0	2.21	0.456	6.06	3.75	81.2	73.9	39.7	31.1	0.190	*nd*	*nd*
30	2.55	0.506	6.89	4.04	121.0	102.9	65.6	41.5	0.239	7.23	7.8
40	2.65	0.518	7.13	4.12	134.4	112.3	74.4	45.0	0.248	7.50	5.5
45	2.68	0.522	7.27	4.17	141.7	117.1	79.5	46.7	0.254	7.70	9.1
50	2.74	0.531	7.41	4.22	150.5	122.8	85.6	48.7	0.261	7.79	13.4
55	2.79	0.539	7.58	4.29	160.6	129.9	92.1	51.3	0.265	7.92	19.8
60	2.85	0.546	7.71	4.30	169.2	134.2	99.0	52.7	0.274	8.07	55.4

The density measurement error was less than 0.01 g/cm^3^. The standard deviation of the sound velocities, elastic moduli, Poisson’s ratio *ν* and Vickers hardness *H*_V_ were within ±0.03 km/s, ±1 GPa, ±0.001 and ±0.06 GPa, respectively. *nd*: not determined.
